# Serotonin Transporter Availability in Early Stage Parkinson's Disease and Multiple System Atrophy

**DOI:** 10.1155/2014/345132

**Published:** 2014-02-03

**Authors:** S. R. Suwijn, H. W. Berendse, C. V. M. Verschuur, R. M. A. de Bie, J. Booij

**Affiliations:** ^1^Department of Neurology, Academic Medical Center, P.O. Box 22660, 1100 DD Amsterdam, The Netherlands; ^2^Department of Neurology, VU University Medical Center, P.O. Box 7057, 1007 MB Amsterdam, The Netherlands; ^3^Department of Nuclear Medicine, Academic Medical Center, P.O. Box 22660, 1100 DD Amsterdam, The Netherlands

## Abstract

*Background*. Differentiating Parkinson's disease (PD) from multiple system atrophy (MSA) can be challenging especially early in the course of the disease. Previous studies have shown that midbrain serotonin transporter (SERT) availability in patients with established MSA was significantly lower compared to PD. It is unknown if this is also true for early-stage patients. *Methods*. 77 early-stage, untreated PD patients were recruited between 1995 and 1998, underwent [^123^I]**β**-CIT SPECT imaging, and were followed for at least five years. 16 patients were lost to followup, and in 4 the diagnosis was changed to another atypical parkinsonian syndrome, but not in MSA. In 50 patients, the PD diagnosis was unchanged at followup. In seven patients, the diagnosis was changed to MSA at followup. We retrospectively assessed baseline midbrain SERT availability as well as midbrain SERT-to-striatal dopamine transporter (DAT) ratios. *Results*. No difference in baseline [^123^I]**β**-CIT SERT availability was found. The midbrain SERT-to-striatal DAT ratio for whole striatum was significantly lower in patients with PD compared to MSA (*P* = 0.049). However, when adjusting for the disease duration at imaging this difference is not significant (*P* = 0.070). *Conclusion*. Midbrain SERT availability is not different between early-stage PD and MSA. Therefore, SERT imaging is not useful to differentiate between early PD and MSA.

## 1. Introduction

A key neuropathological characteristic of Parkinson's disease (PD) is loss of brainstem neurons that produce dopamine [1]. This loss induces features such as bradykinesia and rigidity. The clinical diagnosis of PD is based on the combination of motor features and response to levodopa [2, 3]. Differentiating PD from multiple system atrophy (MSA) can be challenging especially early in the disease course, when signs and symptoms overlap [4, 5]. Indeed, in specialized centers, PD is misdiagnosed in 6–25% of cases [2, 3, 6, 7]. General neurologists misdiagnose PD patients in up to 35% of cases [4]. Dopamine transporter (DAT) imaging with single photon emission computed tomography (SPECT) is reliable in detecting nigrostriatal cell loss. Some studies even suggest that imaging of the DAT can provide more certainty in the differential diagnosis of PD [8, 9], although this is not supported by other studies [10–12].

However, it has become clear that PD is characterized not only by dopaminergic, but also by serotonergic degeneration [13, 14]. Although [^123^I]*β*-carboxymethyoxy-3-*β*-(4-iodophenyl) tropane (*β*-CIT) and its fluoropropyl variant (FP-CIT) are well-known tracers for imaging of the DAT, midbrain binding of these tracers is mainly associated with binding to SERT, while binding in the striatum is mainly associated with DAT [12, 15–19]. Therefore, both DAT and SERT availability can accurately be assessed in the same subject by analysing [^123^I]*β*-CIT binding in different brain areas. The DAT/SERT selectivity is lower for [^123^I]*β*-CIT (1.7 : 1 and 2.8 : 1, resp.), which favours the use of [^123^I]*β*-CIT over [^123^I]FP-CIT to assess extrastriatal SERT binding [20].

Recently, it was shown, using [^123^I]*β*-CIT SPECT, that midbrain SERT availability in patients suffering from MSA is significantly reduced compared to PD [15, 16]. However, since these patients were established cases (mean disease duration of 24.0 months), it is unknown whether midbrain SERT availability is also reduced in early-stage MSA. Therefore, we assessed SERT availability, using [^123^I]*β*-CIT SPECT, in patients with early-stage PD and MSA to determine whether SERT availability can be useful in differentiating early-stage PD from MSA.

## 2. Materials and Methods

### 2.1. Subjects

77 patients with early-stage, untreated PD diagnosed according to the United Kingdom Parkinson's Disease Society Brain Bank criteria [21, 22] and reduced striatal [^123^I]*β*-CIT SPECT binding at the time of the initial clinical diagnosis were recruited at the VU University Medical Center (VUMC) between 1995 and 1998. In seven patients, the diagnosis was changed to MSA during followup, as previously reported [23]. All patients were followed for at least five years to ensure none of the other patients developed signs of MSA or another atypical parkinsonian syndrome. 16 PD patients were lost to followup, two had progressive supranuclear palsy, one had dementia with Lewy bodies, and one had an uncertain diagnosis, leaving 57 patients (50 PD and 7 MSA patients; Table 1) for further analysis.

All subjects underwent [^123^I]*β*-CIT SPECT imaging prior to the initiation of dopaminergic medication. None of the subjects were on medication that could interfere with [^123^I]*β*-CIT SPECT binding (i.e., amphetamine or antidepressants). The medical charts were reviewed to ensure none of the patients were using serotonergic medication. Patients with dementia at baseline were not included; a Mini-Mental State Examination score below 26 was used as an exclusion criterion. At the time of the initial diagnosis the Beck Depression Inventory scale was used to identify and exclude patients with signs of a major depressive disorder. Disease duration was based on the date of the initial diagnosis established by a neurologist. All subjects gave written informed consent to the research protocol, which was approved by the medical ethical committee of the VUMC. The ethical review conformed to the Declaration of Helsinki.

### 2.2. SPECT Imaging

SPECT imaging was performed using a brain-dedicated system, SME 810X system (Strichmann Medical Equipment Inc., Medfield, MA, USA; Neurofocus). This system consists of 12 individual crystals each equipped with a focusing collimator. The spatial resolution of this camera system is approximately 6.5 mm full-width at half-maximum, throughout the 20 cm field of view. [^123^I]*β*-CIT (specific activity >185 MBq/nmol; radiochemical purity >99%) was injected intravenously at an approximate dose of 110 MBq. [^123^I] labelling and acquisition were performed as described previously [24]. Image acquisition was performed 24 hours after injection. Images were corrected for attenuation and reconstructed in 3D [18, 25].

### 2.3. Analysis of Images

For the analysis of striatal and midbrain [^123^I]*β*-CIT binding, representing DAT and SERT binding, respectively, two consecutive transverse slices representing the most intense striatal and midbrain binding were analysed. A standard region of interest (ROI) template (constructed according to a stereotactic atlas) including regions for the caudate nucleus, putamen, whole striatum, midbrain, and occipital cortex (representing nonspecific binding) was placed bilaterally on the images, as previously reported [18]. Estimates of specific midbrain or striatal binding were made by subtracting occipital counts from striatal or midbrain counts. Specific [^123^I]*β*-CIT binding ratio was calculated using the formula (mean binding in ROI-mean occipital binding)/mean occipital binding. This formula is referred to as nondisplaceable binding potential (BP_ND_) [26]. Furthermore, we calculated BP_ND_ in the midbrain versus BP_ND_ assessed in the caudate nucleus, putamen, and striatum (midbrain SERT-to-striatal DAT ratios). All images were analyzed by one operator blinded to the clinical data.

### 2.4. Statistical Analysis

The Kolmogorov-Smirnov test was applied to screen for normality. Midbrain BP_ND_ met with the assumption of normality only after transformation by a natural logarithm. Group differences regarding gender and side of onset were analysed using the chi-square test. Possible differences in age-of-onset, disease duration until imaging, and the UPDRS motor scores were analysed using an independent *t*-test. We compared the [^123^I]*β*-CIT BP_ND_ values and SERT-to-DAT ratios between PD and MSA using ANOVA. Logistic regression was performed to assess the impact of independent predictors. Analysis was done using SPSS 20.0 (IBM Inc., USA) at a significance level of 0.05.

## 3. Results

### 3.1. Patients

The demographic and clinical characteristics are listed in Table 1. The two groups were different in disease duration at imaging (*P* ≤ 0.005) and total followup (*P* ≤ 0.005). There were no differences regarding the other clinical characteristics. 19 patients died during followup (12 PD and all 7 MSA patients).

### 3.2. Analysis of [^123^I]*β*-CIT SPECT Binding

The mean [^123^I]*β*-CIT BP_ND_ was reduced in the putamen compared to the caudate nucleus in all patients. No statistical significant difference in midbrain [^123^I]*β*-CIT BP_ND_ was found between the two groups (Table 2). The mean SERT-to-DAT ratio (midbrain-to-whole striatum) was significantly lower in patients with PD compared to MSA (*P* = 0.048). However, when adjusting significantly different disease duration at imaging this difference was not significant (Table 3).

## 4. Discussion

The results of this study include two important findings; (1) SERT-to-DAT ratios were not different between patients with early PD and MSA. (2) In contrast to a previous report [16], the MSA patients in our study did not have significantly lower midbrain SERT availability compared to PD. This could be explained by the difference in disease duration between the studied populations. Scherfler and colleagues studied PD and MSA patients at a mean disease duration of 20.4 and 24.0 months, respectively. Our population was scanned at a mean disease duration of 12.3 months for PD and 2.4 months for MSA, thus at a very early disease stage. It is possible that serotonergic degeneration in MSA occurs at a different rate at later disease stages compared to PD.

Patients with PD had a longer followup than patients with MSA. This could be explained by the uneven distribution of deceased patients (12 PD and all 7 MSA patients). This was expected considering the mean survival of PD is longer compared to MSA.

Our study has both strengths and limitations. Patients were screened to exclude the presence of major depressive disorders that might have had a possible confounding effect. Furthermore, we reviewed the medical charts, with the current knowledge, to determine whether any of the patients was using a compound that may have interfered with [^123^I]*β*-CIT binding at the time of diagnosis. Moreover, all scans were acquired before any dopaminergic medication was initiated to reduce possible confounding effects. To our knowledge, this is the first study that assesses midbrain SERT availability in prospectively recruited patients with early-stage PD and MSA. Moreover, SPECT imaging was acquired at a mean disease duration of 1.2 years for PD and 0.2 years for MSA, thus at a very early stage.

A possible limitation of the present study is that we used a nonselective tracer to assess both DAT and SERT. Unfortunately, in the period that the patients were recruited and imaged (i.e., 1995–1998) selective SERT tracers, like [^123^I]ADAM, were not available [27]. However, although it is not possible to measure SERT availability in the striatum using [^123^I]*β*-CIT, midbrain [^123^I]*β*-CIT binding is mainly associated with the binding to SERT, while binding in the striatum is mainly associated with binding to DAT [18]. Therefore, DAT and SERT availability can be measured accurately with [^123^I]*β*-CIT SPECT in different areas of the brain. The DAT/SERT selectivity is lower for [^123^I]*β*-CIT (1.7 : 1 and 2.8 : 1, resp.), which favours the use of [^123^I]*β*-CIT over [^123^I]FP-CIT to assess extrastriatal SERT binding [20].

A major limitation is the number of patients with MSA (*n* = 7) in this present study. We can therefore not exclude that in a larger prospective study midbrain SERT availability is significantly lower in early-stage MSA compared to PD. However it is difficult to prospectively recruit MSA patients in a very early stage since these patients are often diagnosed as PD [4, 6, 7]. Therefore large groups of clinically diagnosed PD patients or patients with clinically uncertain parkinsonism will have to be recruited to get a substantial amount of MSA patients imaged at an early stage.

## 5. Conclusions

We found that midbrain SERT availability does not differentiate between PD and MSA patients in an early-stage. Our findings suggest that degeneration of the serotonergic system is similar in the early clinical stages of MSA and PD, but we cannot exclude that degeneration of this system progresses at different rates in later diseases stages. Therefore SPECT imaging targeting the SERT is not useful to differentiate between early PD and MSA.

## Figures and Tables

**Table 1:** Demographic and clinical characteristics.

	PD (*n* = 50)	MSA (*n* = 7)	*P* value
Age-of-onset (mean ± SD), yr	54.4 ± 9.7	56.7 ± 14.7	0.70
Male sex-no. (%)	31 (62.0)	4 (57.1)	0.81
Total followup (mean ± SD), yr	14.2 ± 3.7	6.0 ± 3.9	<0.005
Most affected side (striatal) (right/left)	22/28	3/4	0.96
Disease duration at imaging (mean ± SD), yr	1.2 ± 1.8	0.2 ± 0.14	<0.005
UPDRS motor score at imaging (0–108) (mean ± SD)	19.0 ± 6.2	20.4 ± 5.6	0.56

PD: Parkinson's disease, MSA: multiple system atrophy, SD: standard deviation, and UPDRS: unified parkinson's disease rating scale.

**Table 2:** Mean specific to nonspecific [^123^I]*β*-CIT midbrain binding ratio (BP_ND_) (mean ± SD) and midbrain SERT-to-striatal DAT ratios.

Region of interest	PD (*n* = 50)	MSA (*n* = 7)	*P*-value
Midbrain	1.03 ± 0.37	1.21 ± 0.38	0.28
Striatum, whole	4.08 ± 0.27	4.15 ± 0.42	0.93
Caudate nucleus	5.79 ± 1.84	6.38 ± 2.67	0.78
Putamen	3.15 ± 0.97	3.41 ± 1.33	0.52
Midbrain/putamen	0.36 ± 0.13	0.40 ± 0.11	0.44
Midbrain/caudate nucleus	0.18 ± 0.06	0.22 ± 0.06	0.09
Midbrain/striatum	0.26 ± 0.09	0.34 ± 0.14	0.048

SERT: serotonin transporter, DAT: dopamine transporter, SD: standard deviation, PD: Parkinson's disease, and MSA: multiple system atrophy.

**Table 3:** Independent predictors of diagnosis.

	Constant	Midbrain-to- striatum ratio	Disease duration at imaging
*β*	−3.161	7.285	−2.614
*P*-value	0.020	0.070	0.237

*β*: logistic regression coefficient.
